# 1-Ethenyl-4-[(phenylsulfanyl)methyl]­benzene

**DOI:** 10.1107/S1600536812002899

**Published:** 2012-02-10

**Authors:** Alcives Avila-Sorrosa, Reyna Reyes-Martínez, Simón Hernández-Ortega, David Morales-Morales

**Affiliations:** a Instituto de Química, Universidad Nacional Autónoma de México, Circuito exterior, Ciudad Universitaria, México DF 04510, Mexico

## Abstract

The dihedral angle between the aromatic rings in the title compound, C_15_H_14_S, is 72.38 (7)°. In the crystal, the mol­ecules are connected by C—H⋯π inter­actions.

## Related literature

For aryl­sulfides used as ligands in coordination chemistry, see: Olivos-Suárez *et al.* (2007 ▶); Fierro-Arias *et al.* (2005 ▶).
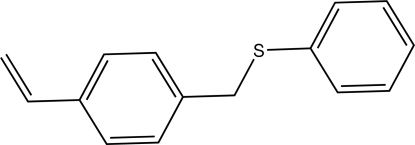


## Experimental

### 

#### Crystal data

C_15_H_14_S
*M* *_r_* = 226.32
Monoclinic, 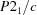

*a* = 8.3042 (19) Å
*b* = 14.642 (3) Å
*c* = 10.370 (2) Å
β = 92.912 (4)°
*V* = 1259.3 (5) Å^3^
*Z* = 4
Mo *K*α radiation
μ = 0.23 mm^−1^
*T* = 298 K
0.36 × 0.19 × 0.10 mm

#### Data collection

Bruker SMART APEX CCD diffractometer
Absorption correction: multi-scan (*SADABS*; Bruker, 2007 ▶) *T*_min_ = 0.936, *T*_max_ = 0.982
10072 measured reflections
2314 independent reflections
1525 reflections with *I* > 2σ(*I*)
*R*_int_ = 0.034

#### Refinement

*R*[*F* ^2^ > 2σ(*F* ^2^)] = 0.057
*wR*(*F* ^2^) = 0.140
*S* = 1.03
2314 reflections
145 parameters
1 restraint
H-atom parameters constrained
Δρ_max_ = 0.27 e Å^−3^
Δρ_min_ = −0.13 e Å^−3^

### 

Data collection: *SMART* (Bruker, 2007 ▶); cell refinement: *SMART*; data reduction: *SAINT* (Bruker, 2007 ▶); program(s) used to solve structure: *SHELXS97* (Sheldrick, 2008 ▶); program(s) used to refine structure: *SHELXL97* (Sheldrick, 2008 ▶); molecular graphics: *SHELXTL* (Sheldrick, 2008 ▶); software used to prepare material for publication: *SHELXTL*.

## Supplementary Material

Crystal structure: contains datablock(s) I, global. DOI: 10.1107/S1600536812002899/bt5794sup1.cif

Structure factors: contains datablock(s) I. DOI: 10.1107/S1600536812002899/bt5794Isup2.hkl

Supplementary material file. DOI: 10.1107/S1600536812002899/bt5794Isup3.cml

Additional supplementary materials:  crystallographic information; 3D view; checkCIF report

## Figures and Tables

**Table 1:** Inter­molecular C—H⋯π inter­actions in the title compound (Å)

H atom	Centroid	Distance	Symmetry code
H9	C1–C6	2.746	(−*x* + 1, −*y* + 1, −*z*)
H12	C1–C6	2.873	(−*x*, *y* + 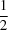 , −*z* + 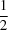 )

**Fig. 1.**
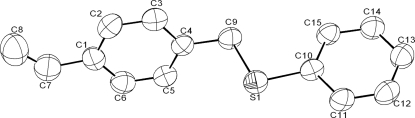
Crystal structure of the title compound with the numbering scheme. Displacement ellipsoids are shown at the 30% probability. H atoms have been omitted for clarity.

**Fig. 2.**
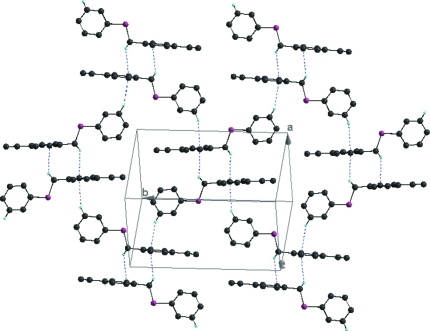
C—H—π dimeric motif, by C9—H9B—Cg and two-dimensional sheet structure formed through C—H—π interactions, C12—H12—Cg.

## supplementary crystallographic information

### Comment

Organosulfur compounds are important in synthetic organic chemistry, their applications include protecting groups (thioacetals), reversing of the polarity (Umpolong), in enhancement of the acidity of C—H bonds, as well as in transfer of chirality from sulfur to carbon, among many other. Additionally, sulfur-containing groups are frequently found in important drugs used in the treatment of various diseases like diabetes, Alzheimer's, Parkinson's, cancer, and HIV. Thus, given their potential applications of these species, the synthesis of these compounds including catalytic procedures for their efficient production, has become an area of growing interest, this being particularly true for the attaining of non-symmetric sulfides. Moreover, recently arylsulfides have been used successfully as ligands in coordination chemistry (Olivos-Suárez *et al.*, 2007) where simple variations of the substitution at the sulfur makes them a very interesting set of ligands for the fine tuning of electron-donor properties and thus modulating the metal reactivity (Fierro-Arias *et al.*, 2005).

The molecular structure of the title compound is showed in Fig. 1. The bond distances and angles are within normal values. The geometry of the molecule exhibits non-coplanarity of the phenyl rings, with a dihedral angle of 72.38 (7)°. The molecules are stabilized in the solid state by weak C—H-π*Cg*(C1—C6) intermolecular interactions, [C9—H9B—*Cg* 2.746 Å, C9—*Cg* 3.488 Å, C9—H9—*Cg* 132.9°, symm. code -*x* + 1, -*y* + 1, -*z*, C12—H12—*Cg* 2.873 Å, C12—*Cg* 3.651 Å, C12—H12—*Cg*, 141.9° symm operator 2]. The C—H-π interaction between the methylene group and the aromatic ring *Cg* of the molecules generates a dimeric motif which are extended by C—H-π interaction between C12—H12–*Cg* generating a two-dimensional sheet structure (Fig. 2).

### Experimental

To a suspension of NaH (126 mg, 5.5 mmol) in 20 ml of THF, benzenethiol was added dropwise (0.34 ml, 5 mmol). The resulting suspension was stirred for 10 min and after this time chloromethyl-vinyl-benzene (this starting material was used as a mixture 60:40% of 1-chloromethyl-3-vinyl-benzene and 1-chloromethyl-4-vinyl-benzene as supplied by Aldrich Chemical Co., 0.7 ml, 760 mg, 5 mmol) was added. The reaction mixture was then allowed to proceed under stirring for further 3 h. Upon completion the reaction mixture was extracted with CH_2_Cl_2_ (4 × 20 ml) and the combined organic fractions were washed with H_2_O (2 × 50 ml) and dried with Na_2_SO_4_, filtered and evaporated under vacuum to afford 1.10 g (4.84 mmol, 97%) of a white solid consisting in a mixture of a 1-phenylsulfanylmethyl-3-vinyl-benzene and 1-phenylsulfanylmethyl-4-vinyl-benzene (60:40 *ca*). Crystals suitable for X-ray analysis were obtained from a slow evaporation of a saturated solution of this mixture in CH_2_Cl_2_. IR (KBr): 3087, 3003, 2921, 2848, 1588, 1509, 1488, 1091, 993, 907, 851, 820, 627 cm^-1^. EM–IE: 226 (40, [*M*+]), 117 (100), 115 (30) / 226 (15, [*M*+]), 117 (100), 115 (20) m/*z* (%). ^1^H NMR (300 MHz, CDCl_3_), d (p.p.m.): 7.37–7.17 (m, 18H) 6.73 (dd, 1H), 6.68 (dd, 1H), 5.74 (dd, 2H), 5.26 (dd, 2H), 4.12 (s, 2H), 4.13 (s, 2H).

### Refinement

H atoms were included in calculated positions (C—H = 0.93 Å), and refined using a riding model with *U*_iso_(H) = 1.2*U*_eq_ of the carrier atom.

### Crystal data

**Table:**

C_15_H_14_S	*F*(000) = 480
*M**_r_* = 226.32	*D*_x_ = 1.194 Mg m^−^^3^
Monoclinic, *P*2_1_/*c*	Mo *K*α radiation, λ = 0.71073 Å
Hall symbol: -P 2ybc	Cell parameters from 2634 reflections
*a* = 8.3042 (19) Å	θ = 2.4–24.2°
*b* = 14.642 (3) Å	µ = 0.23 mm^−^^1^
*c* = 10.370 (2) Å	*T* = 298 K
β = 92.912 (4)°	Block, colourless
*V* = 1259.3 (5) Å^3^	0.36 × 0.19 × 0.10 mm
*Z* = 4	

### Data collection

**Table:**

Bruker SMART APEX CCD diffractometer	2314 independent reflections
Radiation source: fine-focus sealed tube	1525 reflections with *I* > 2σ(*I*)
Graphite monochromator	*R*_int_ = 0.034
Detector resolution: 0.661 pixels mm^-1^	θ_max_ = 25.4°, θ_min_ = 2.4°
ω scans	*h* = −10→10
Absorption correction: multi-scan (*SADABS*; Bruker, 2007)	*k* = −17→17
*T*_min_ = 0.936, *T*_max_ = 0.982	*l* = −12→12
10072 measured reflections	

### Refinement

**Table:**

Refinement on *F*^2^	Primary atom site location: structure-invariant direct methods
Least-squares matrix: full	Secondary atom site location: difference Fourier map
*R*[*F*^2^ > 2σ(*F*^2^)] = 0.057	Hydrogen site location: inferred from neighbouring sites
*wR*(*F*^2^) = 0.140	H-atom parameters constrained
*S* = 1.03	*w* = 1/[σ^2^(*F*_o_^2^) + (0.0603*P*)^2^ + 0.2262*P*] where *P* = (*F*_o_^2^ + 2*F*_c_^2^)/3
2314 reflections	(Δ/σ)_max_ < 0.001
145 parameters	Δρ_max_ = 0.27 e Å^−^^3^
1 restraint	Δρ_min_ = −0.13 e Å^−^^3^

### Special details

**Table:**

Geometry. All e.s.d.'s (except the e.s.d. in the dihedral angle between two l.s. planes) are estimated using the full covariance matrix. The cell e.s.d.'s are taken into account individually in the estimation of e.s.d.'s in distances, angles and torsion angles; correlations between e.s.d.'s in cell parameters are only used when they are defined by crystal symmetry. An approximate (isotropic) treatment of cell e.s.d.'s is used for estimating e.s.d.'s involving l.s. planes.
Refinement. Refinement of *F*^2^ against ALL reflections. The weighted *R*-factor *wR* and goodness of fit *S* are based on *F*^2^, conventional *R*-factors *R* are based on *F*, with *F* set to zero for negative *F*^2^. The threshold expression of *F*^2^ > σ(*F*^2^) is used only for calculating *R*-factors(gt) *etc*. and is not relevant to the choice of reflections for refinement. *R*-factors based on *F*^2^ are statistically about twice as large as those based on *F*, and *R*-factors based on ALL data will be even larger.

### Fractional atomic coordinates and isotropic or equivalent isotropic displacement parameters (Å^2^)

**Table:**

	*x*	*y*	*z*	*U*_iso_*/*U*_eq_	
S1	0.16819 (9)	0.60414 (5)	0.19138 (7)	0.0904 (3)	
C1	0.3712 (4)	0.2798 (2)	0.0931 (3)	0.0885 (8)	
C2	0.2757 (4)	0.3174 (2)	−0.0058 (3)	0.0930 (9)	
H2	0.2263	0.2793	−0.0676	0.112*	
C3	0.2521 (3)	0.4107 (2)	−0.0150 (3)	0.0885 (8)	
H3	0.1867	0.4343	−0.0824	0.106*	
C4	0.3246 (3)	0.4690 (2)	0.0748 (3)	0.0757 (7)	
C5	0.4190 (4)	0.4312 (2)	0.1723 (3)	0.0886 (8)	
H5	0.4685	0.4691	0.2343	0.106*	
C6	0.4426 (4)	0.3392 (3)	0.1813 (3)	0.0974 (9)	
H6	0.5087	0.3161	0.2486	0.117*	
C7	0.4016 (5)	0.1808 (3)	0.1094 (4)	0.1230 (12)	
H7	0.4742	0.1640	0.1765	0.148*	
C8	0.3390 (6)	0.1155 (3)	0.0413 (5)	0.1552 (17)	
H8A	0.2656	0.1284	−0.0270	0.186*	
H8B	0.3670	0.0553	0.0603	0.186*	
C9	0.2985 (3)	0.5699 (2)	0.0659 (3)	0.0873 (8)	
H9A	0.2493	0.5855	−0.0180	0.105*	
H9B	0.4009	0.6015	0.0764	0.105*	
C10	0.1452 (3)	0.7229 (2)	0.1694 (2)	0.0752 (7)	
C11	0.0489 (3)	0.7681 (2)	0.2549 (3)	0.0869 (8)	
H11	0.0009	0.7354	0.3195	0.104*	
C12	0.0245 (4)	0.8600 (2)	0.2446 (3)	0.0995 (10)	
H12	−0.0404	0.8891	0.3024	0.119*	
C13	0.0937 (4)	0.9101 (2)	0.1508 (3)	0.1001 (9)	
H13	0.0764	0.9727	0.1446	0.120*	
C14	0.1886 (4)	0.8663 (2)	0.0666 (3)	0.0981 (9)	
H14	0.2363	0.8997	0.0025	0.118*	
C15	0.2150 (3)	0.7736 (2)	0.0749 (3)	0.0860 (8)	
H15	0.2801	0.7450	0.0166	0.103*	

### Atomic displacement parameters (Å^2^)

**Table:**

	*U*^11^	*U*^22^	*U*^33^	*U*^12^	*U*^13^	*U*^23^
S1	0.0786 (5)	0.0978 (6)	0.0979 (6)	0.0027 (4)	0.0353 (4)	0.0027 (4)
C1	0.0791 (19)	0.089 (2)	0.100 (2)	0.0046 (16)	0.0288 (17)	0.0090 (19)
C2	0.0802 (19)	0.102 (2)	0.098 (2)	−0.0160 (17)	0.0154 (17)	−0.0222 (19)
C3	0.0724 (17)	0.109 (2)	0.0849 (19)	0.0017 (16)	0.0074 (15)	−0.0013 (18)
C4	0.0635 (15)	0.0884 (19)	0.0771 (17)	−0.0015 (14)	0.0203 (13)	−0.0026 (16)
C5	0.0860 (19)	0.100 (2)	0.0804 (19)	−0.0012 (17)	0.0075 (16)	−0.0066 (17)
C6	0.091 (2)	0.113 (3)	0.089 (2)	0.0079 (19)	0.0086 (17)	0.005 (2)
C7	0.122 (3)	0.122 (3)	0.129 (3)	−0.006 (2)	0.034 (2)	−0.002 (3)
C8	0.161 (4)	0.125 (4)	0.183 (5)	−0.010 (3)	0.039 (4)	0.005 (3)
C9	0.0807 (18)	0.098 (2)	0.0854 (18)	0.0025 (15)	0.0248 (14)	−0.0026 (16)
C10	0.0550 (13)	0.0940 (19)	0.0771 (16)	−0.0007 (13)	0.0100 (12)	−0.0057 (14)
C11	0.0699 (16)	0.104 (2)	0.0886 (19)	0.0054 (15)	0.0247 (14)	−0.0012 (16)
C12	0.088 (2)	0.114 (3)	0.098 (2)	0.0153 (19)	0.0257 (18)	−0.014 (2)
C13	0.101 (2)	0.095 (2)	0.105 (2)	0.0097 (18)	0.0141 (19)	−0.0066 (19)
C14	0.108 (2)	0.096 (2)	0.093 (2)	−0.0036 (18)	0.0259 (18)	0.0007 (18)
C15	0.0818 (18)	0.095 (2)	0.0832 (18)	−0.0009 (15)	0.0231 (15)	−0.0068 (16)

### Geometric parameters (Å, º)

**Table:**

S1—C10	1.763 (3)	C8—H8A	0.9300
S1—C9	1.805 (3)	C8—H8B	0.9300
C1—C6	1.374 (4)	C9—H9A	0.9700
C1—C2	1.379 (4)	C9—H9B	0.9700
C1—C7	1.481 (5)	C10—C15	1.380 (4)
C2—C3	1.382 (4)	C10—C11	1.390 (3)
C2—H2	0.9300	C11—C12	1.364 (4)
C3—C4	1.379 (4)	C11—H11	0.9300
C3—H3	0.9300	C12—C13	1.368 (4)
C4—C5	1.365 (4)	C12—H12	0.9300
C4—C9	1.495 (4)	C13—C14	1.366 (4)
C5—C6	1.363 (4)	C13—H13	0.9300
C5—H5	0.9300	C14—C15	1.377 (4)
C6—H6	0.9300	C14—H14	0.9300
C7—C8	1.282 (4)	C15—H15	0.9300
C7—H7	0.9300		
			
C10—S1—C9	104.23 (13)	C4—C9—S1	108.70 (19)
C6—C1—C2	117.0 (3)	C4—C9—H9A	110.0
C6—C1—C7	118.6 (3)	S1—C9—H9A	110.0
C2—C1—C7	124.4 (4)	C4—C9—H9B	110.0
C1—C2—C3	121.3 (3)	S1—C9—H9B	110.0
C1—C2—H2	119.3	H9A—C9—H9B	108.3
C3—C2—H2	119.3	C15—C10—C11	118.2 (3)
C4—C3—C2	120.6 (3)	C15—C10—S1	125.1 (2)
C4—C3—H3	119.7	C11—C10—S1	116.6 (2)
C2—C3—H3	119.7	C12—C11—C10	120.5 (3)
C5—C4—C3	117.6 (3)	C12—C11—H11	119.8
C5—C4—C9	121.5 (3)	C10—C11—H11	119.8
C3—C4—C9	120.9 (3)	C11—C12—C13	121.2 (3)
C6—C5—C4	121.8 (3)	C11—C12—H12	119.4
C6—C5—H5	119.1	C13—C12—H12	119.4
C4—C5—H5	119.1	C14—C13—C12	118.7 (3)
C5—C6—C1	121.6 (3)	C14—C13—H13	120.7
C5—C6—H6	119.2	C12—C13—H13	120.7
C1—C6—H6	119.2	C13—C14—C15	121.2 (3)
C8—C7—C1	127.2 (5)	C13—C14—H14	119.4
C8—C7—H7	116.4	C15—C14—H14	119.4
C1—C7—H7	116.4	C14—C15—C10	120.2 (3)
C7—C8—H8A	120.0	C14—C15—H15	119.9
C7—C8—H8B	120.0	C10—C15—H15	119.9
H8A—C8—H8B	120.0		
			
C6—C1—C2—C3	−0.6 (4)	C3—C4—C9—S1	107.0 (3)
C7—C1—C2—C3	179.8 (3)	C10—S1—C9—C4	−179.3 (2)
C1—C2—C3—C4	0.4 (4)	C9—S1—C10—C15	0.1 (3)
C2—C3—C4—C5	−0.3 (4)	C9—S1—C10—C11	−179.8 (2)
C2—C3—C4—C9	−179.5 (2)	C15—C10—C11—C12	0.2 (4)
C3—C4—C5—C6	0.4 (4)	S1—C10—C11—C12	−179.9 (2)
C9—C4—C5—C6	179.7 (3)	C10—C11—C12—C13	−0.1 (5)
C4—C5—C6—C1	−0.7 (4)	C11—C12—C13—C14	0.0 (5)
C2—C1—C6—C5	0.7 (4)	C12—C13—C14—C15	0.0 (5)
C7—C1—C6—C5	−179.6 (3)	C13—C14—C15—C10	0.0 (5)
C6—C1—C7—C8	176.0 (4)	C11—C10—C15—C14	−0.1 (4)
C2—C1—C7—C8	−4.4 (6)	S1—C10—C15—C14	180.0 (2)
C5—C4—C9—S1	−72.3 (3)		

### Intermolecular C—H···π interactions in the title compound (Å)

**Table:**

H atom	Centroid	Distance	Symmetry code
H9	C1–C6	2.746	(-x+1, -y+1, -z)
H12	C1–C6	2.873	(-x, y+1/2, -z+1/2)
